# Right atrial and SVC infiltrating mass-the entity of infiltrating lipoma

**DOI:** 10.1186/s13019-019-1015-7

**Published:** 2019-12-02

**Authors:** Owais A. Shah, Abdul Badran, Markku Kaarne, Theodore Velissaris

**Affiliations:** 1 St. George’s Hospital Medical School, University of London, London, UK; 2 Department of Cardiothoracic Surgery, University Hospital Southampton, Southampton, UK

**Keywords:** Infiltrating cardiac lipoma, Cardiac leiomyosarcoma, Right atrial tumour

## Abstract

**Background:**

Cardiac lipomas are rare benign primary cardiac tumours primarily composed of mature adipocytes. They are usually well defined, encapsulated masses, but rarely demonstrate malignant characteristics by infiltrating the myocardium. This causes diagnostic uncertainty as it becomes a priority to rule out primary malignant cardiac tumours such as sarcoma which often carry a poor prognosis.

**Case Report:**

A 61 year old female presenting with chest pain was found to have an infiltrating right atrial hypertrophic mass. Mutli-disciplinary team (MDT) discussions along with the presence of symptoms and likelihood of malignancy led to the recommendations for surgery.

Intraoperatively this involved the right pulmonary veins and superior vena cava (SVC). The mass was resected with good margins and reconstruction of the right atrium, pulmonary veins and SVC was done using porcine pericardial patch. The patient made a good postoperative recovery and was discharged home in sinus rhythm with no significant valvular lesions. This was further confirmed at 6 month follow up. Final histology was that of infiltrating lipoma.

**Conclusions:**

In this rare case of infiltrating cardiac lipoma in a relatively young patient, the diagnostic uncertainty despite multimodal imaging meant surgery was indicated due to the high suspicion of cancer. Even in benign cases, fatty infiltration can lead to conduction defects and embolisation. Technical difficulties in sectioning these specimens is caused by intra-tumour variability and current recommendations are for excision biopsy, for best characterisation.

The management of these patients requires an MDT with Cardiac surgery being a safe approach providing definitive management.

## Background

Primary cardiac neoplasms are rare with post-mortem incidence estimates ranging from 0.001% to 0.03% [1]. Three quarters of these lesions are benign with most being cardiac myxomas. Other benign lesions include tumours of adipose tissue such as lipoma and lipomatous hypertrophy which together account for 6-10% cases. Cardiac lipomas are well defined encapsulated benign masses composed predominantly of mature adipocytes. They can however infiltrate into the underlying myocardium in which case they are known as infiltrating cardiac lipoma [2]. Cardiac lipomas are most frequently observed in the right atrium and left ventricle however they can arise in numerous locations within the heart with 50% being subendocardial, 25% pericardial and 25% intramyocardial [3, 4]. They can occur at any age with no gender predisposition reported [3]. Cardiac lipomas are usually incidental findings remaining asymptomatic however they can result in syncope, embolisation and obstruction of blood flow within the chambers or the coronary arteries due to mass effect.

Furthermore, myocardial infiltration can result in conduction defects causing rhythm disturbances [5].

## Case Report

A 61 year old female with a background of asthma, hypertension and previous smoking, presented with chest pain and was found to have an infiltrating right atrial hypertrophic mass suspicious for sarcoma on transthoracic echocardiography and cardiac MRI.

**Fig. 1:**
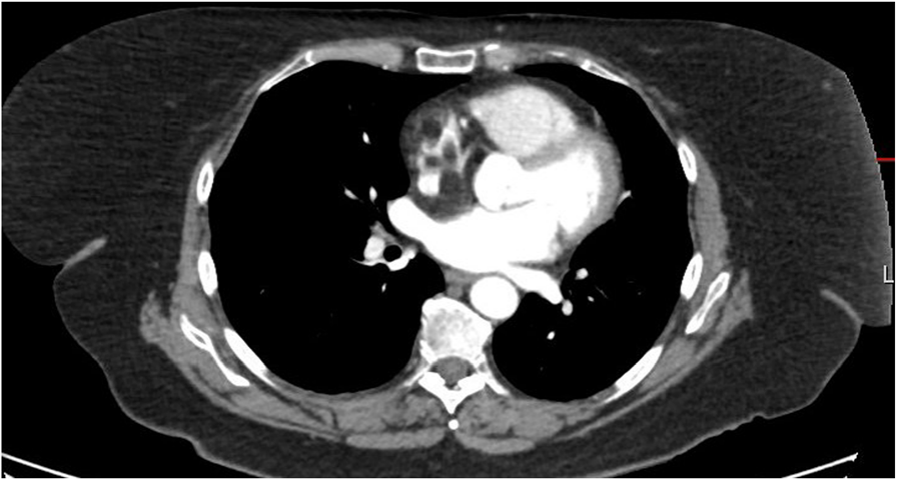
Transverse plane contrast CT thorax showing right atrial mass

**Fig. 2:**
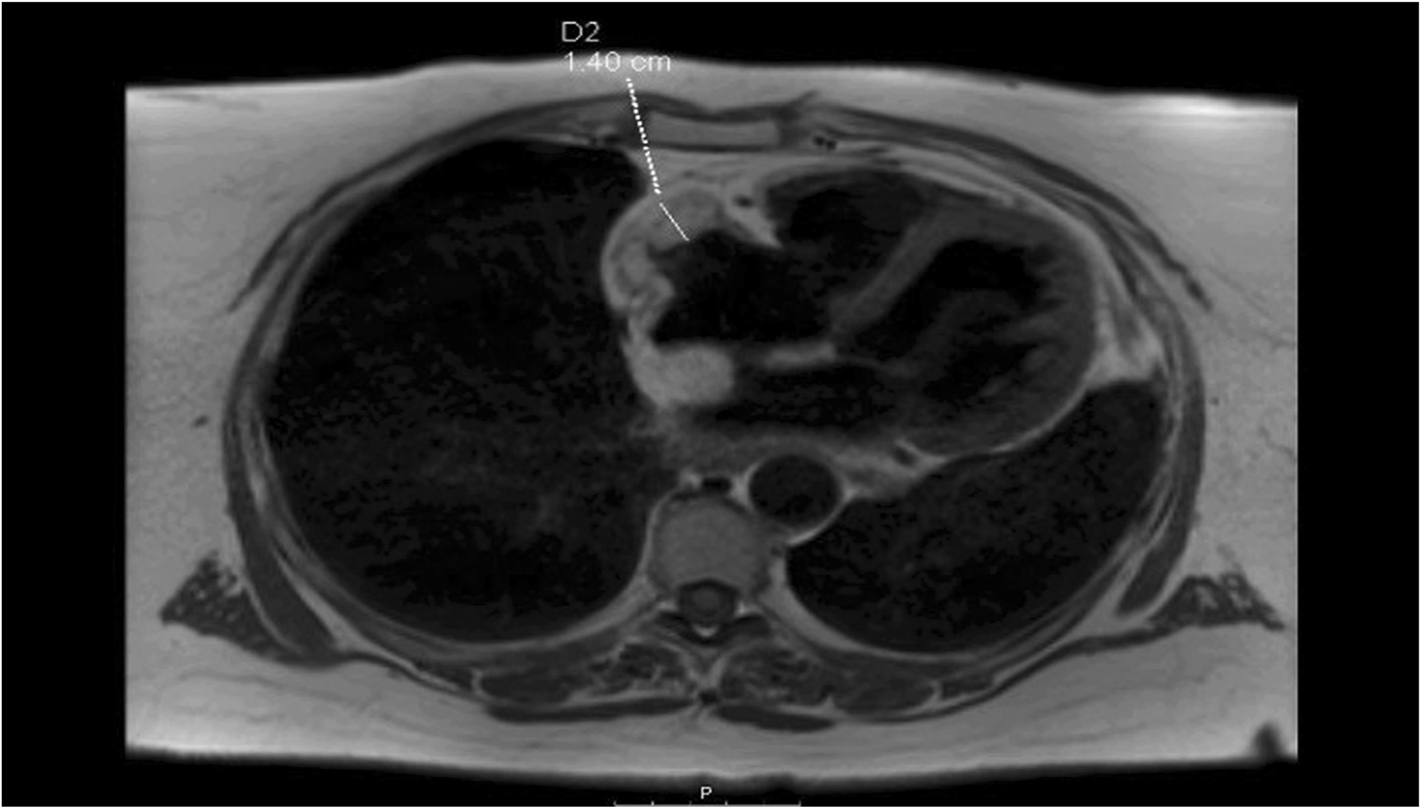
Transverse T1 weighted Cardiac MRI showing right atrial mass

**Fig. 3:**
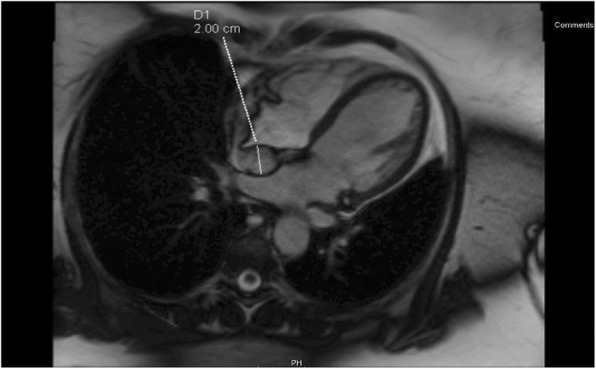
Transverse T2 weighted Cardiac MRI showing right atrial mass

Coronary angiogram showed no flow-limiting lesions and cardiac echocardiogram showed restrictive changes with epicardial thickening in the right atrium and around the superior vena cava (SVC). Cardiac computed tomography (CT)(Fig. 1) and magnetic resonance imaging (MRI)(Figs. 2 and 3) revealed an infiltrative fatty lesion encased in the SVC, invading into the right atrium.

Following multi-disciplinary team (MDT), patient and family discussions, the patient consented to surgery.

Intraoperatively, there was a fatty infiltrating mass involving the right atrium, right superior pulmonary vein and SVC. The patient was placed onto cardiopulmonary bypass using bicaval cannulation with the SVC cannulated as distal as possible. The mass was resected with good margins. Right atrium, pulmonary veins and proximal SVC were reconstructed using porcine pericardium.

Tissue was sent for histology, Sections showed lipomatous tumour entrapping myocardial cells, composed of lobules of mature adipocytes and very thin fibro-vascular septa. No significant cytological atypia was observed. On molecular genetic testing, MDM2 gene amplification was negative by interphase FISH ruling out a malignant liposarcoma. A diagnosis of infiltrating lipoma involving the right atrium was established.

The patient made a good postoperative recovery and was discharged in sinus rhythm with no significant valvular lesions. This was confirmed at six months review.

## Discussion and Conclusions

Infiltrating cardiac lipoma provides a diagnostic challenge. It cannot be distinguished from primary malignant cardiac lesions such as liposarcoma based on clinical presentation or non- invasive diagnostic modalities. Cardiac liposarcomas are aggressive malignant mesenchymal neoplasms which carry poor prognosis and hence represent important differentials to be ruled out [6]. Intra-cavitary lipomas usually present as homogenous hyperechoic masses on echocardiography however these findings are not diagnostic and hence cannot be utilised to conclusively distinguish cardiac lipomas from cardiac liposarcomas [7]. Both cardiac computerized tomography (CT) and cardiac MRI can identify fat and hence aid tissue characterisation. Studies have shown cardiac MRI to be the gold standard diagnostic imaging modality for cardiac lipoma. This typically shows a high signal intensity on T1 weighted images which decreases drastically with fat suppression signals [7, 8]. Unfortunately, even MRI has limited sensitivity, helping to distinguish 69% of cases in the setting of well differentiated liposarcoma [9]. This uncertainty was demonstrated when assessing the MRI of our patient where in light of the fatty, infiltrative nature of the lesion, we could not conclusively rule out liposarcoma. Conclusive diagnosis requires histological analysis of tissue specimen. Furthermore, due to sampling errors and intra-tumour variability, accurate analysis and best tissue characterisation requires excision biopsy [10].

In a literature review, we found seven cases of infiltrating cardiac lipoma. Three of these cases describe lipomas originating on the epicardial aspect infiltrating into the underlying ventricular myocardium one of which reported multiple infiltrating lipomas [11–13]. The remaining four cases report intercavitary tumours. Of these, one case reported right ventricular lipoma involving the interatrial septum and tricuspid valve [2], one case reported bi-ventricular tumour invading the underlying myocardium [14], one was that of a left atrial mass extending to the ventricles [15] and one case reported an infiltrating lipoma involving the mitral valve [16]. Presenting complaints in these reports included palpitations, presyncope, paroxysms of tachycardia, collapse, dyspnoea and anasarca. Surgical resection of the tumour was reported and advocated in five of the cases. Of the remaining two cases, one described a post mortem case [11] while the other reported extensive recurrent infiltrating lipoma where complete resection was not originally possible due to the close proximity of vital structures [14].

Due to the rarity of cardiac lipomas, there have been no large cohort studies or randomised trials to produce guidelines on treatment. Prompt surgical management can however lead to complete cure resulting in excellent long-term prognosis. Even if benign, infiltrating cardiac lipomas can manifest in fatal clinical sequelae such as arrythmias and heart failure. The management of infiltrating lipomas provides for diagnostic challenges and hence requires an MDT approach and comprehensive patient counselling. We have demonstrated cardiac surgery in this setting is safe and provides good medium to long-term results.

## Data Availability

Not applicable

## Abbreviations

CT: Computed Tomography
FISH: Fluorescence in situ hybridization
MDT: Multi-Disciplinary Team
MRI: Magnetic Resonance Imaging
SVC: Superior Vena Cava
